# An HLAII-targeted DNA vaccine against influenza H7N9 protected mice and ferrets from a virus challenge

**DOI:** 10.1038/s41541-025-01341-4

**Published:** 2025-12-24

**Authors:** Rineke de Jong, Fan Zhou, Tor Kristian Andersen, Norbert Stockhofe-Zurwieden, Rebecca Jane Cox, Bjarne Bogen, Gunnveig Grodeland

**Affiliations:** 1 Wageningen University and Research, Wageningen Bioveterinary Research, Lelystad, The Netherlands; 2 Influenza Centre, Department of Clinical, University of Bergen, Bergen, Norway; 3 Institute of Clinical Medicine, University of Oslo, Oslo, Norway; 4 Department of Microbiology, Haukeland University Hospital, Bergen, Norway; 5 Department of Immunology and Transfusion Medicine, Oslo University Hospital, Oslo, Norway; 6 Center for Pandemics and One-Health Research, SUSTAINIT, Institute of Health and Society, University of Oslo, Oslo, Norway

**Keywords:** Diseases, Immunology, Microbiology

## Abstract

Due to antigenic drift and shift, influenza A viruses may cause new future pandemics. Currently used seasonal influenza vaccines are of little use against novel viruses with pandemic potential. Genetic vaccines can be rapidly produced and could therefore mitigate pandemic outbreaks. Here, we present preclinical proof of protective efficacy of a DNA vaccine encoding a vaccine protein that targets influenza hemagglutinin (HA) to human leukocyte antigen class II (HLAII) molecules on antigen presenting cells (APC). Vaccination of mice raised robust levels of neutralizing antibodies, and protection against a lethal challenge with influenza H7N1 virus. In ferrets, we observed induction of neutralizing antibodies and T-cell responses after a single vaccination, with levels increasing after a second dose. Protection of vaccinated ferrets against a viral challenge with influenza H7N9 was dose dependent, with ferrets receiving the highest vaccine dose being completely protected from clinical disease. In sum, these results warrant progression to a human clinical Phase I trial.

## Introduction

The recent pandemic caused by SARS-CoV-2 has reminded us that it is difficult to predict which virus will constitute the next pandemic threat. As such, it is key to develop efficient vaccine platforms that rapidly can be updated and deployed to counter the next global threat. As demonstrated with the first approval of mRNA vaccines for societal use in 2021^1,2^, genetic vaccines are versatile and easy to scale up. However, the cold chain requirement for current mRNA vaccines may present an obstacle to global deployment. In that respect, DNA vaccines may have an advantage since they are stable across a wide range of temperatures, and a first DNA vaccine for human use against SARS-CoV-2 has been approved in India^3^.

Previously, we have developed a DNA vaccine format designed to rapidly raise protective immune responses against influenza^4–6^. Efficacy was enhanced by targeting of the antigen to selected receptors on antigen presenting cells (APC)^7,8^. Importantly, the APC receptor to which the antigen was targeted could selectively polarize the type of elicited immune responses. As an example, targeting of antigen to major histocompatibility class II (MHCII) molecules rapidly induced an immune response characterized by high antibody titers, whereas targeting towards different chemokine receptors more polarized responses towards T cell activation and cytotoxic T cell responses^6,9,10^. Since neutralizing antibodies are key for protection against influenza, we developed a targeting moiety that binds human leukocyte antigen class II (HLAII) molecules on APC of all humans^11,12^. Importantly, the HLAII-specific targeting moiety cross-reacted to MHCII molecules in several large mammals, thus enabling preclinical evaluation of an HLAII-targeted vaccine against influenza H1N1 in ferrets, pigs, and rhesus macaques^11^.

In March 2013, zoonotic transmission of avian influenza A H7N9 was reported to the World Health Organization (WHO)^13^. Since then, over 1500 laboratory confirmed human infections have been reported, with a case fatality rate of about 39%^14^. Since 2003, there have also been repeated outbreaks of influenza H5N1, with more recent transmissions in farmed minks^15^, as well as outbreaks in cats, red foxes, and fur animal farms^16^. At present, avian influenza H5N1 infections in cows and chickens are threatening human health. Should H5N1 or H7N9 influenza acquire the ability of efficient human-to-human transmission^17,18^, we may face a pandemic with severe consequence. Therefore, we should prepare for the likely future scenario of emerging new influenza viruses with pandemic potential, to which the population only have limited protection. Clearly, it is key to have available efficient vaccine platforms that rapidly can be adapted to counter new pandemic threats.

Here, we present preclinical data for an HLAII-targeted vaccine against influenza A/Shanghai/2/2013 (H7N9). Importantly, the HLAII-targeted vaccine platform has been designed to facilitate easy antigenic updates, thus enabling vaccines to effectively match future emerging influenza viruses. The vaccine platform is based on DNA, which has typically been hampered by reduced efficacy after translation to larger animals and humans^19^. We here found that selective targeting of the major influenza surface protein hemagglutinin (HA) to HLAII molecules on APCs rapidly induced neutralizing antibodies and T-cell responses in HLAII-transgenic mice and ferrets, the latter considered the gold standard animal model for human influenza. Importantly, vaccinated ferrets were protected against a viral challenge with the homologous influenza H7N9 virus. Therefore, the HLAII-targeted vaccine format could represent an important tool for protecting humans against the next emerging influenza virus with a pandemic potential.

## Results

### MHCII-targeted DNA vaccines induced antibodies and protection against influenza H7N1 in mice

**Fig. 1:**
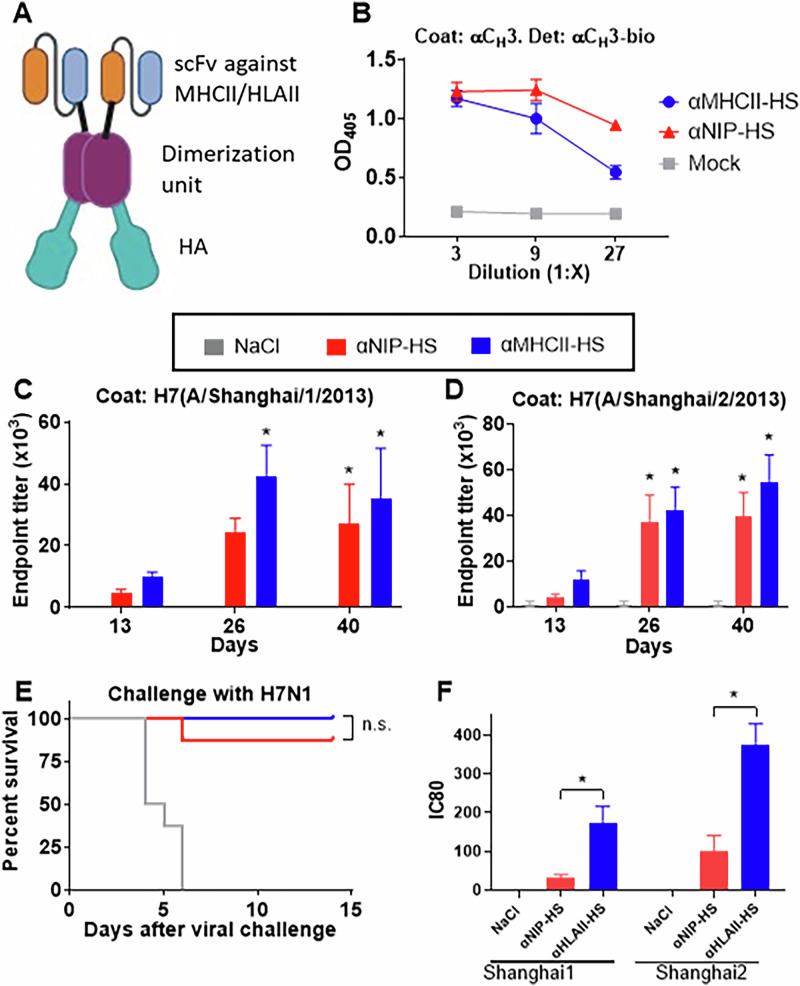
Vaccine induced protection against H7N1 in mice. **A** Following DNA vaccination, cells at the site of injection will secrete vaccine proteins consisting of (i) a targeting unit (scFv) specific for either MHCII or HLAII molecules or the corresponding non-targeted control scFv against the hapten NIP, (ii) a dimerization unit derived from the hinge and C_H_3 domain of hIgG3, and (iii) influenza hemagglutinin (HA). Created in BioRender. **B** Supernatants from transiently transfected 293E cells were evaluated for vaccine protein expression by ELISA with the indicated coat and detection mAbs. **C**-**E** BALB/c mice (n = 8/group), were vaccinated once i.d. with 25 µg DNA encoding the indicated vaccines. Sera from individual vaccinated mice were for the indicated timepoints analyzed for antibodies against A/Shanghai/1/2013 (H7N9) (**C**) and A/Shanghai/2/2013 (H7N9) (HS) in ELISA (**D**). *p < 0.05 as compared to NaCl by Two-Way ANOVA and Tukey’s multiple comparison test. **E** At day 42 after vaccination, mice were challenged with a 5xLD50 dose of influenza A/turkey/Italy/3889/1999 (H7N1) virus. Depicted is survival as defined by the humane endpoint (20% weight loss) (see Supplementary Fig. 1 for weight loss). **F** DQ2 transgenic mice (*n* = 8 mice/group) were vaccinated once i.d. and sera collected by cardiac puncture at day 45 post vaccination for evaluation in a pseudotype neutralization assay against A/Shanghai/1/2013 (H7N9) and HS. Further, A/Vietnam/1203/2004 (H5N1) was included as a negative control virus (not shown). *p < 0.05 as compared to corresponding NIP by the Kruskal–Wallis test.

Plasmids containing the MHCII-targeted vaccine format encode a bivalent fusion protein comprising (i) a targeting unit consisting of a mAb-derived single chain fragment variable (scFv) specific for either mouse MHCII (I-E^d^) molecules or human leukocyte antigen class II (HLAII) molecules, (ii) a dimerization unit derived from the hinge and C_H_3 domain of human IgG3, and (iii) an antigenic unit consisting of influenza HA from influenza A/Shanghai/2/2013 (H7N9) (HS)^5,8^. Following plasmid uptake and translation, transfected cells will secrete a vaccine protein that targets HA to MHCII molecules on APC (Fig. 1A). In addition to the MHCII-targeted vaccines, we constructed similar vaccines where the targeting moiety was replaced with a scFv specific for the hapten NIP (non-targeted control vaccine).

HA from influenza A/Shanghai/2/2013 (H7N9) (HS) was inserted into a pLNOH2^20^ plasmid expression cassette encoding the MHCII-targeted vaccine format (αMHC-HS), as well as non-targeted control vaccine plasmid where the targeting moiety had been replaced with a scFv specific for the hapten NIP (αNIP-HS). Transient transfections with the constructed vaccines confirmed efficient protein expression in vitro (Fig. 1B).

BALB/c mice were vaccinated once with the different vaccine plasmids, and antibody responses against A/Shanghai/1/2013 (H7N9) and HS were evaluated in sera obtained at different time points after vaccination. HA from A/Shanghai/1/2013 (H7N9) and HS are of different origins^21^, and share a 98.44% identify. Interestingly, antibody responses against A/Shanghai/1/2013 (H7N9) were significantly increased at days 26 and 40 in mice receiving αMHCII-HS, but responses after vaccination with αNIP-HS were only significantly elevated at day 40 (Fig. 1C). By contrast, both αMHCII-HS and αNIP-HS induced significant amounts of antibodies against the vaccine encoded strain HS at days 26 and 40 (Fig. 1D).

At week 6 post vaccination, these same mice were challenged with a lethal dose of influenza A/turkey/Italy/3889/1999 (H7N1) virus. This virus is heterologous to HS, with an HA identity of 96.5%. We observed that all mice vaccinated with αMHCII-HS were protected against the challenge, whereas 1 of 8 vaccinated with αNIP-HS had to be euthanized (Fig. 1E). These results showed cross-protection between different H7 strains (Fig. 1C–E)^22^, despite all the mice displaying an initial weight loss prior to recovery (Supplementary Fig. 1).

We proceeded with vaccination of DQ2 transgenic mice with a vaccine specific for HLAII molecules (αHLAII-HS), or the non-targeted control vaccine αNIP-HS. Importantly, αHLAII-HS significantly elicited more potent neutralizing antibodies against both A/Shanghai/1/2013 (H7N9) and HS than did αNIP-HS (Fig. 1F). Neither of the vaccines induced neutralizing antibodies against A/chicken/Italy/13474/1999 (H7N1), A/chicken/Chile/4322/2002 (H7N3), or A/Vietnam/1203/2004 (H5N1) (data not shown).

In sum, vaccination with either αMHCII-HS or αNIP-HS significantly raised antibody responses against the homologous HS in mice and protected against a lethal challenge with a heterologous H7 influenza virus. However, αMHCII-HS induced significantly more antibodies against HA from the heterologous A/Shanghai/1/2013 virus, and αHLAII-HS had a clear advantage over αNIP-HS for induction of neutralizing antibody responses. Based on these promising data, we decided to initiate progression towards evaluation of αHLAII-HS in humans.

### Selection of optimized vaccine plasmids for human use

**Fig. 2:**
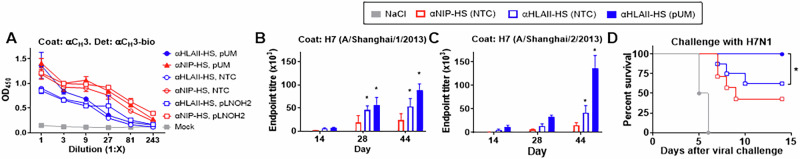
Vector comparison in mice. **A** Supernatants from transiently transfected 293E cells were evaluated in triplicates for protein expression by ELISA with mAbs against the C_H_3-based dimerization unit both for coating and detection. **B**–**D** DQ2 transgenic mice (*n* = 6–8/group) were vaccinated once i.d. with 25 µg DNA encoding the indicated vaccines. Sera from individual vaccinated mice were evaluated for IgG at different timepoints in ELISA against A/Shanghai/1/2013 (H7N9) (**B**) and HS (**C**). *p < 0.05 as compared to NaCl with Two-Way ANOVA and Tukey’s multiple comparison test. **D** At day 47 post vaccination, mice were challenged with a 5xLD50 dose of influenza A/turkey/Italy/3889/1999 (H7N1) virus. Depicted is survival as defined by the humane endpoint (20% weight loss). *p < 0.05 for αHLAII-HS (NTC) vs αHLAII-HS (pUM) with Mantel-Cox test.

A key to successful DNA vaccination is efficient protein production by transfected cells at the site of delivery. This is needed both to enable antigen recognition by B and T cells, as well as utilize the immune-enhancing effect of APC-targeting by the MHCII-/HLAII-specific targeting moieties^5,11^. Thus, we inserted αHLAII-HS and αNIP-HS into the pUMVC^23^ and NTC7482^24^ plasmids, each optimized for protein expression and translation to clinical use. Transient transfections in vitro demonstrated comparable secretion efficacy to that of pLNOH2 (a plasmid developed for efficient antibody production in vitro^20^) in HEK293E cells (Fig. 2A). Interestingly, αNIP-HS was consistently secreted at a higher level as compared to αHLAII-HS for all plasmids, although the difference was not statistically significant.

DQ2-transgenic mice were vaccinated once with pUMVC and NTC7482 plasmids encoding αHLAII-HS, or NTC7482 encoding αNIP-HS. IgG antibody levels against HA from influenza A/Shanghai/1/2013 and HS were significantly increased after a single vaccination with either of the αHLAII-HS vaccines, whereas the same was not observed for αNIP-HS (Fig. 2B, C). At day 47 after vaccination, mice were challenged with a lethal dose of influenza A/turkey/Italy/3889/1999 (H7N1) virus. Vaccination with αHLAII-HS in the pUMVC vector significantly improved protection as compared to αHLAII-HS in NTC7482 (Fig. 2, D). This experiment was repeated with similar results (Supplementary Fig. 2).

In sum, all the plasmids secreted vaccine proteins in vitro to similar levels. However, following vaccination of mice there was a clear tendency of improved antibody induction with αHLAII-HS encoded in pUMVC as compared to NTC7482, as well as significantly improved survival after viral challenge. Thus, progression to larger animals and humans should use the pUMVC plasmid.

### Safety and antibody induction after vaccination with pUMVC encoded αHLAII-HS in ferrets

**Fig. 3:**
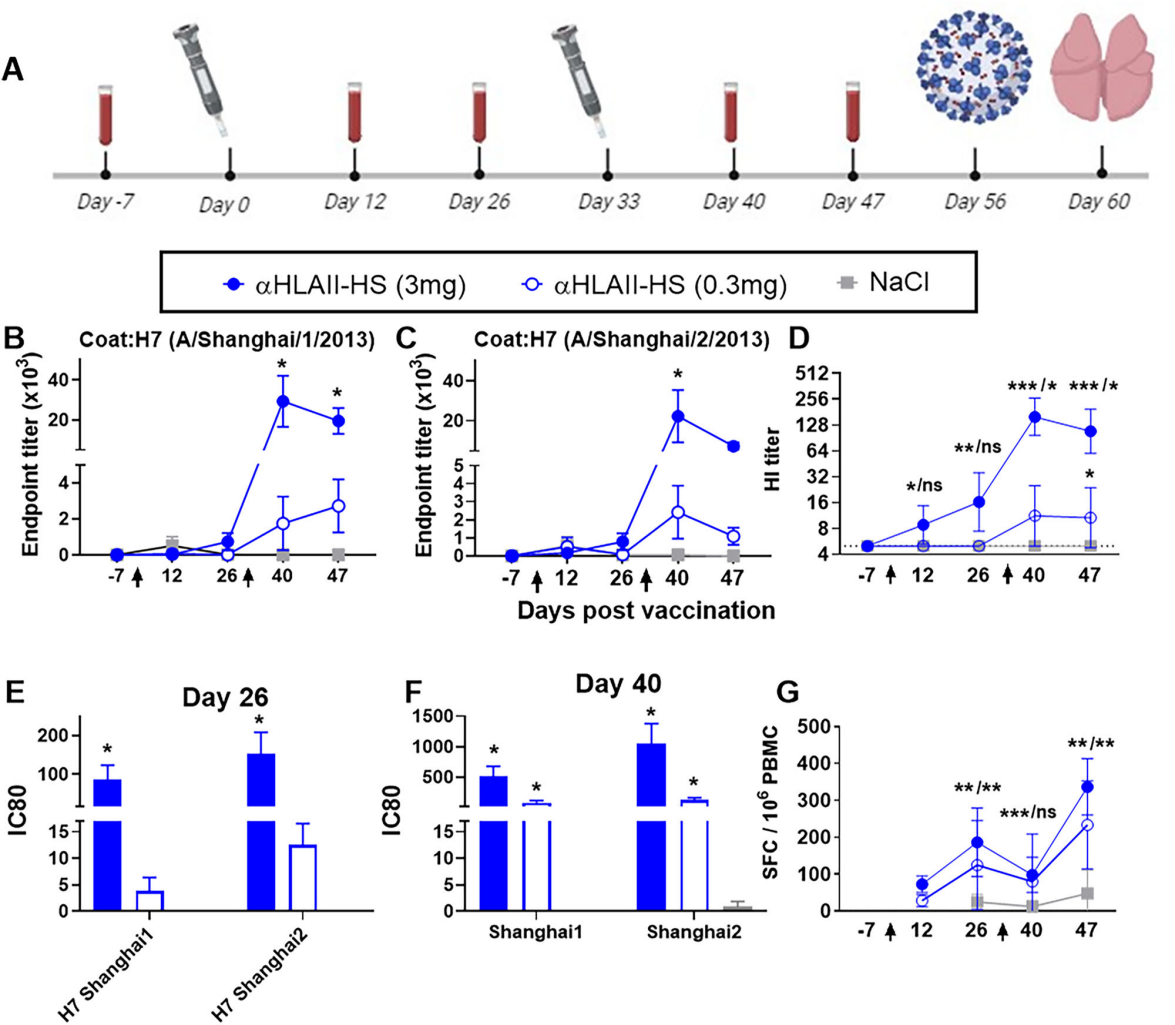
Antibody responses after vaccination of ferrets. **A** Study design: Ferrets were allocated to three groups (*n* = 8/group) and DNA vaccinated twice at days 0 and 33 with jet injections intradermally. Blood samples were collected longitudinally, as indicated, for evaluations of antibody and T-cell responses. At day 56, ferrets were challenged with A/Anhui/1/2013 (H7N9) influenza virus. At day 60, the experiment was ended and animals necropsied. IgG in sera from vaccinated ferrets was evaluated individually at different timepoints in ELISA against HA from A/Shanghai/1/2013 (H7N9) (**B**), and HS (**C**). *p < 0.0003 for αHLAII-HS (3 mg) vs. αHLAII-HS (0.3 mg) and NaCl, Two-Way ANOVA and Tukey’s multiple comparison test. **D** Geometric mean (±95% CI) haemagglutination inhibition (HI) titers (reciprocal values) in sera against inactivated whole virus influenza A/Anhui/1/2013 virus. Kruskal–Wallis per timepoint followed by Wilcoxon test for pair-wise comparison, *p < 0.05, **p < 0.01, ***p < 0.001, as compared to NaCl. Left of “/ “ = results for αHLAII-HS (3 mg), right results of αHLAII-HS (0.3 mg). Sera after one (**E**) and two DNA vaccinations (**F**) were evaluated in a pseudotype neutralization assays against A/Shanghai/1/2013 (H7N9) and A/Shanghai/2/2013 (H7N9). *p < 0.02 as compared to NaCl, One-Way ANOVA, and Tukey’s multiple comparison test. **G** Number of spot-forming cells (SFC) in IFNγ ELISpot (mean ± 95% CI) after in vitro stimulation with inactivated A/Anhui/1/2013 (H7N9) influenza virus. *n* = 8/group, except for the 3 mg αHLAII-HS (*n* = 5) and 0.3 mg αHLAII-HS (*n* = 2) vaccinated ferrets on day 12. Further, on day 40, *n* = 6 for 0.3 mg αHLAII-HS, and *n* = 7 for NaCl. Kruskal Wallis per timepoint followed by Wilcoxon test for pair-wise comparison: ns: non-significant. **p < 0.01 as compared to NaCl. Left of “/ “ = results of αHLAII-HS (3 mg), right results of αHLAII-HS (0.3 mg).

Ferrets represent the gold standard model for preclinical influenza vaccine research. Thus, we set up a dose finding experiment in ferrets to evaluate safety, immunogenicity and efficacy following two vaccinations with αHLAII-HS (Fig. 3A). Previously, we have demonstrated that gentler needle-free intradermal delivery was at least as efficient as needle injection followed by electroporation for vaccination of larger animals against H1N1 influenza^11^. As such, ferrets were here vaccinated intradermally by the needle-free infection system Tropis®. Importantly, no fever was recorded post vaccination in either of the three groups (Supplementary Fig. 3A). On the first day post vaccination (days 1 and 34), local mild to occasionally moderate skin redness was observed in all ferrets in the group vaccinated with 3.0 mg αHLAII-HS, and in one and four ferrets post first and second dose with 0.3 mg αHLAII-HS, respectively. In ferrets vaccinated with NaCl, redness was observed in one animal after the first dose, and in two ferrets after the second. For all groups, the skin reaction was transient and had disappeared on day two post vaccination (days 2 and 35) (Supplementary Fig. 3B).

The first vaccination did not significantly increase serum IgG levels above those of the NaCl-treated group, but the second vaccination boosted responses against both A/Shanghai/1/2013 and HS influenza viruses (Fig. 3B, C). No antibody responses were observed against HA from the negative control A/Vietnam/1203/2004 (H5N1) influenza virus (not shown).

For a more qualitative assessment of the vaccine induced antibodies, an HI-assay was set up against the homologous influenza virus A/Anhui/1/2013 (H7N9) (HA from A/Anhui/1/2013 virus is homologous to HS). HI titers were significantly enhanced after one vaccination with 3 mg of αHLAII-HS, while for 0.3 mg a booster dose was required (Fig. 3D). This result was confirmed in a pseudotype neutralization assays performed against HS (Fig. 3E, F). In this assay, we also evaluated responses against a panel of selected pseudotype influenza H7 viruses. Similar to the quantitative ELISA evaluation (Fig. 3B, C), a significant increase in neutralizing antibodies against the heterologous A/Shanghai/1/2013 influenza virus was observed after the first dose, but not against the more distant H7 influenza strains evaluated (Fig. 3E, F, and Supplementary Fig. 4). The second vaccination also boosted some neutralizing antibodies against A/chicken/Chile/4322/2002 (H7N3), A/chicken/Italy/13474/1999 (H7N1), and A/Guandong/17SF003/2016 (H7N9), albeit not statistically significant (sequence alignments in Supplementary Fig. 5).

### Induction of cellular immunity after vaccination of ferrets

While the vaccine induced neutralizing antibodies may potentially prevent viral infection, their efficacy is typically reduced by antigenic drift. Such evolution is expected to also occur when a new influenza virus with pandemic potential emerges, so it is therefore important to evaluate if a pandemic vaccine can raise broader types of immunity. Thus, we stimulated PBMC with either HA protein from influenza HS or inactivated whole virus from homologous A/Anhui/1/2013 (H7N9) influenza, and evaluated interferon γ (IFNγ) secretion as an indication of T-cell activation (Fig. 3G and Supplementary Fig. 6). On day 12, we saw a slight increase in T-cell responses, but few animals were evaluated at this time point. Starting from day 26, we observed a significant increase in cellular immunity for both vaccine doses. The booster dose given at day 33 further boosted responses, after an initial decrease in responses observed a week post the second dose. Mean spot counts were consistently higher in the group vaccinated with 3.0 mg as compared to 0.3 mg αHLAII-HS, but this difference was not statistically significant.

### Vaccination protected ferrets against a viral challenge with influenza H7N9

**Fig. 4:**
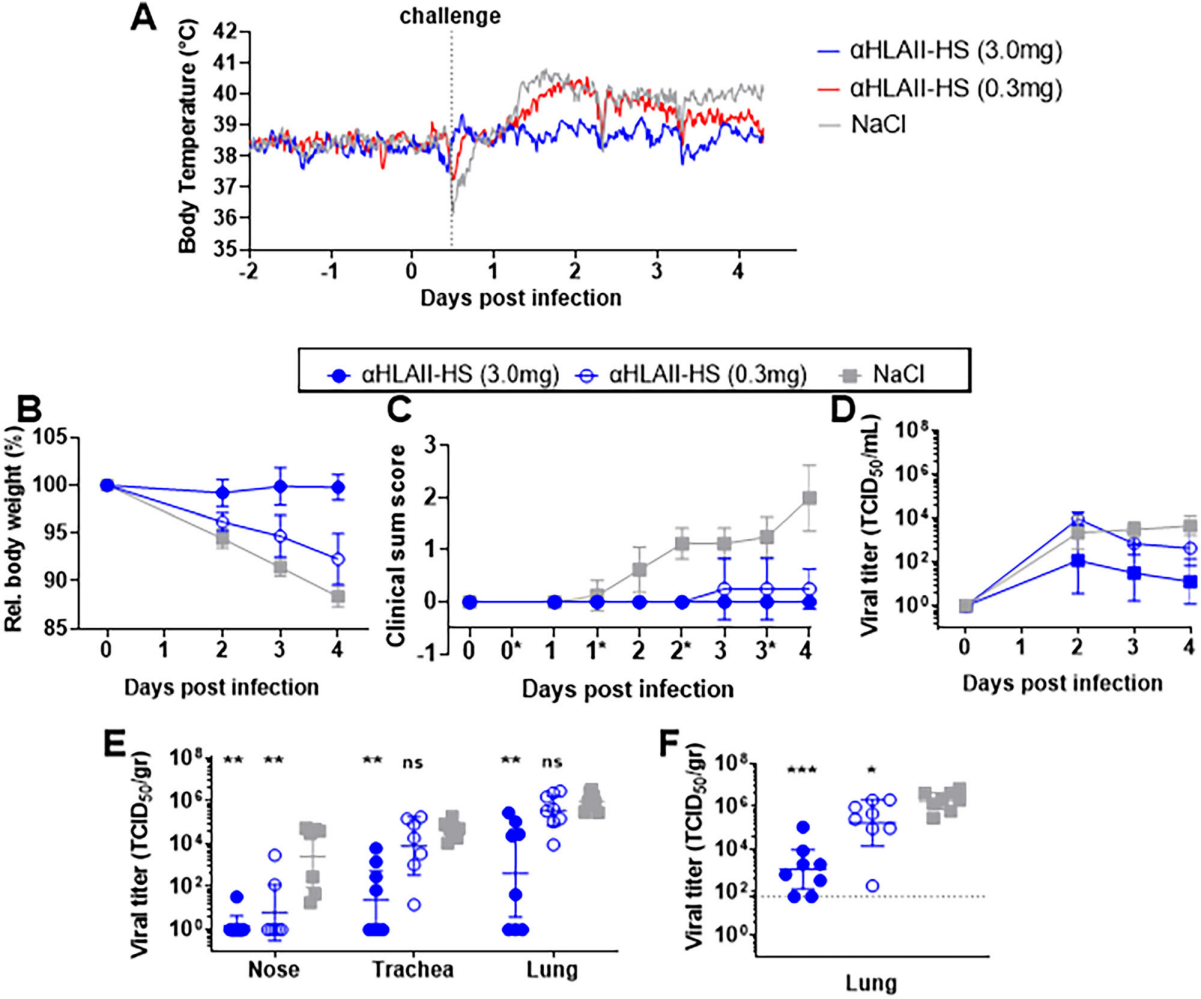
Protection and reduced virus replication following a viral challenge with influenza H7N9 in vaccinated ferrets. Ferrets were challenged with influenza A/Anhui/1/2013 (H7N9) and monitored for clinical signs of disease (*n* = 8 per group). **A** Mean body temperature based on continuous measurements. Due to the malfunctioning of one transponder, *n* = 7 for the 3.0 mg αHLAII-HS group. See Supplementary Fig. 8 for individual values. **B** Mean body weight (±95% CI), where values are expressed as percentages related to the body weight prior to challenge (defined as 100%). **C** Mean clinical sum score (±95% CI) based on observations twice daily (* on *x*-axis indicates afternoon session), including depression, nasal discharge and respiratory distress. See Supplementary Fig. 8 for scoring of clinical signs. **D** PCR equivalent viral titers in throat swabs. Shown is geometric mean (±95% CI). **E** PCR equivalent titers (TCID_50_/gr) in tissue samples from nasal turbinates, trachea, and lung at necropsy on day 4 post viral infection. Shown is geometric mean (±95% CI). Kruskal–Wallis per timepoint followed by Wilcoxon test for pair-wise comparison, ***p < 0.001, as compared to NaCl, **p < 0.01, ***p < 0.001, ns: non significant. For nasal turbinates n = 7 of 0.3 mg αHLAII-HS and NaCl vaccinated ferrets; for trachea *n* = 7 of 0.3 mg αHLAII-HS vaccinated ferrets. **F** Viral titers in lung homogenates analysed by end-point titration on MDCK cells. Based on tissue weights, viral titers were calculated as TCID_50_ / gram tissue. Geometric mean (±95% CI) is shown. Kruskal–Wallis per timepoint followed by Wilcoxon test for pair-wise comparison, *p < 0.05, ***p < 0.001, as compared to NaCl.

At day 56, 23 days post a second vaccination, ferrets were challenged with a highly pathogenic dose of the homologous influenza A/Anhui/1/2013 (H7N9) influenza virus. The sedation needed for viral challenge led to a slight decrease in temperature for all groups around this timepoint. However, from about one day post viral challenge, the saline vaccinated ferrets developed pronounced fever (Fig. 4A and Supplementary Fig. 7) accompanied by body weight losses varying from 9 to 14% on day 4 post viral challenge (Fig. 4B), as well as mild to moderate depression, watery nasal discharge, and slightly increased breathing frequencies (Fig. 4C. See Supplementary fig. 8 for scoring of clinical signs). Vaccination showed a dose-dependent protective efficacy on the clinical outcomes. The ferrets vaccinated with 0.3 mg αHLAII-HS developed substantial fever, but generally recovered faster from the clinical signs of disease as compared to saline treated controls. In contrast, vaccination with 3.0 mg αHLAII-HS protected the ferrets from fever, weight loss, as well as clinical signs of disease.

For an assessment of viral replication, we evaluated throat, nasal turbinates, trachea, and lung tissues at day 4 post infection. All tissues from saline vaccinated ferrets tested positive for virus. The highest infectious titers were found in the lungs (10^6^ TCID50 per gram tissue), while lower and more variable viral loads were found in nasal turbinate samples (Fig. 4D–F). Importantly, vaccination with αHLAII-HS consistently reduced viral replication in all tissues. For the 0.3 mg αHLAII-HS vaccine group, we observed only a minor reduction of viral loads in the throat, trachea, and lung tissue samples, but viral loads were statistically significantly reduced in nasal turbinate tissues. Following vaccination with 3.0 mg αHLAII-HS we observed a reduced viral replication in the throat and significantly lower infectious titers in the nose, trachea, and lungs. Interestingly, 2 out of 8 ferrets in this group were completely protected, with no viral RNA or infectious virus detected in any of the samples.

**Fig. 5:**
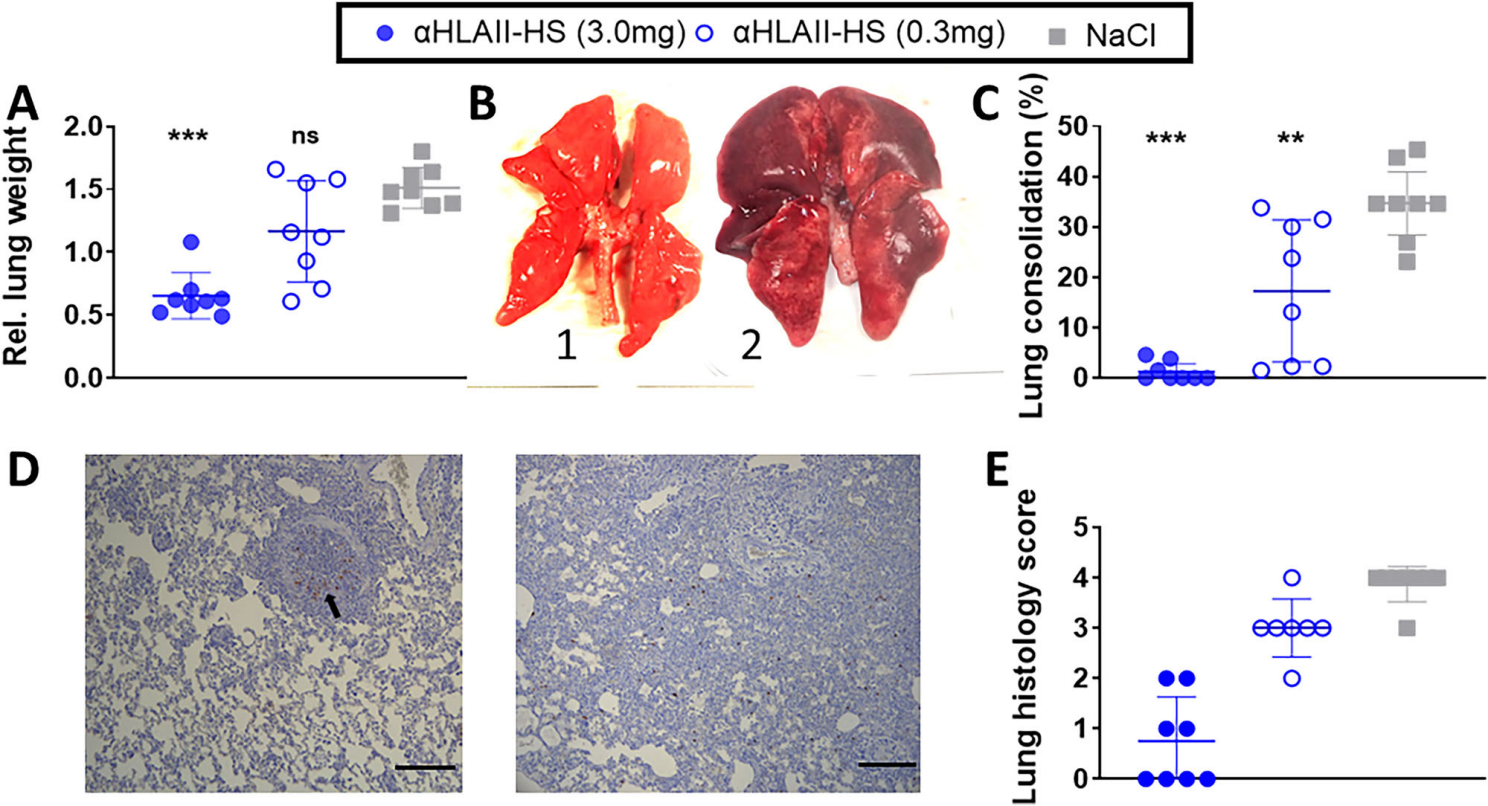
Reduced lung pathology following a viral challenge with influenza H7N9 virus in vaccinated ferrets. Four days post viral infection, ferrets (*n* = 8/group) were necropsied. Lungs were removed and evaluated for **A** mean (SD) relative lung weight. Values were calculated as percentages related to the body weight on the day of challenge. Kruskal–Wallis followed by Wilcoxon test for pair-wise comparison, ***p < 0.001, as compared to NaCl. **B** Photos of representative lungs; 1: normal healthy lung (αHLAII-HS 3.0 mg vaccinated); 2: pneumonic lung with extended dark-red consolidations in all lobes (NaCl vaccinated ferret). **C** Mean (SD) percentages of macroscopic lung tissue consolidation. Kruskal–Wallis per timepoint followed by Wilcoxon test for pair-wise comparison, **p < 0.01, ***p < 0.001, as compared to NaCl. **D** Photos of representative histopathology and immunohistochemistry (IHC) for influenza A viral antigen; Left panel (αHLAII-HS, 3 mg): Focal endobroncitis and accumulation of mononuclear and neutrophils in the bronchial space and IHC positive staining of endobronchial epithelium (arrow). Right panel (NaCl): Extended bronchopneumonia with alveolar edema and extended cell infiltrations with neutrophils, lymphocytes, and macrophages in the alveolar space; IHC positive cells multifocal disseminated in the alveolar tissue of the lungs. Obj. magnification: 10x. Scale bars = 100 µm. **E** Mean (SD) score of histological changes ranging from score 0 (no changes) to score 4 (severe changes).

The dose dependent protection observed (Fig. 4) was confirmed also upon pathological lung examinations. In the group vaccinated with 3.0 mg of αHLAII-HS, the mean relative lung weight was 0.65%, which was significantly different from the αHLAII-HS group vaccinated with 0.3 mg (1.17%) and the saline treated control (1.51%) group (Fig. 5A). The viral infection caused severe pneumonia in all saline vaccinated ferrets, with multifocal or coalescing dark-red consolidations (Fig. 5B). By contrast, 6 of 8 lungs in the group vaccinated with 3.0 mg αHLAII-HS had no gross changes, and small foci were recorded only in two lungs. For the group vaccinated with 0.3 mg αHLAII-HS, 5 out of 8 ferrets had extended lung lesions, and focal changes were observed in 3 lungs. The mean areas of consolidations were significantly reduced in both vaccine groups (1.25 ± 1.93% in 3.0 mg of αHLAII-HS vaccine group, and 17.3 ± 14.12% in the 0.3 mg vaccine group), as compared to the saline treated control group (34.71 ± 7.50%) (Fig. 5C).

Histopathology revealed focal endobronchitis lesions (scores 1 or 2) in 4 lungs from ferrets vaccinated with 3 mg αHLAII-HA, and increasingly extended lesions with endobronchitis and alveolar cell degeneration, alveolar edema and neutrophil infiltration in the alveolar space in the 0.3 mg vaccinated group (score 2–4). The most extended lesions and severity were observed in the saline vaccinated control group (Fig. 5D). The mean histology score was significantly lower in the group vaccinated with 3.0 mg αHLAII-HS, as compared to the saline vaccinated group. No significant differences in histology scores were observed between the 0.3 mg group and the saline vaccinated group (Fig. 5E). Immunohistochemical staining of influenza A viral (IAV) antigen was restricted to few positive spots in the bronchial epithelium in 4 of 8 lungs in the 3 mg αHLAII-HS group, whereas in the saline and the 0.3 mg vaccinated group, positive staining was observed in bronchial epithelium and in the alveolar epithelium and other alveolar cells (Fig. 5B, E). The mean IHC score of the 3 mg vaccinated group [mean score 0.75 (SD 0.88)] differed significantly (*p* < 0.01) from the mean score of the 0.3 vaccinated group [mean score 2.1 (SD 0.9)] and the control group [mean score 3.0 (SD 0.5)].

In sum, we observed good protection from an influenza H7N9 viral challenge in ferrets vaccinated with 3.0 mg αHLAII-HS, but only partial protection in ferrets vaccinated with 0.3 mg αHLAII-HS.

## Discussion

It is improbable that we in advance will be able to accurately predict the emergence of the next influenza virus with a pandemic potential. Currently, a variant of H5N1 could acquire the ability of human transmission due to the frequent zoonotic infections observed in different geographical sites, as well as the mass spread in wild birds and mammals^25–27^, but history warns that pandemic predictions are extremely difficult^28^. As such, it is key to have available vaccine formats that are highly versatile and can be updated for protection against whichever viral variant will represent a future pandemic threat.

Previously, we have demonstrated a protective potential in mice, ferrets and pigs following insertion of HA from influenza H1N1 into the MHCII-targeted vaccine format^5,11^, as well as in mice for HA from A/chicken/Italy/13474/99 (H7N1) and A/turkey/Italy/3889/1999 (H7N1) influenza viruses^4^. Importantly, we have also confirmed functional antibody induction against 6 different subtypes of influenza^29^. Here, we subcloned HA from the potential pandemic strain of influenza HS into HLAII-targeted vaccines, with the aim to translate results into a human vaccine. The experiments demonstrated induction of both T-cell responses and neutralizing antibodies even after a single DNA vaccination in ferrets, and a dose dependent protection against a viral challenge with influenza H7N9 after two vaccinations. Importantly, ferrets in the 3.0 mg dose group were completely protected against clinical signs of disease, whereas ferrets receiving 0.3 mg were partially protected, indicating a dose-effect. The low dose group also displayed reduced virus excretion and strongly reduced pathology in the respiratory tract, which indicates at least some potential for reduction of viral transmission. As for safety, vaccination was generally well tolerated in the ferrets, with increasing frequencies of transient and mild adverse events in the highest dose group, as expected.

How will the evaluated doses translate to humans? We have previously observed significant antibody induction after HLAII-targeted vaccination against influenza H1N1 with 25 µg DNA in Norwegian farm pigs. We did not perform corresponding dose titrations in ferrets, but found that a single dose with 100 µg of DNA robustly raised neutralizing antibodies against influenza H1N1^11^. For the present ferret study, we found that higher doses were needed. Due to the apparent differences in immunogenicity between vaccination of ferrets with influenza H1N1 as compared to here H7N9, the likely explanation is variable immunogenicity inherent to HA from different subtypes of influenza. Interestingly, influenza H7N9 has consistently been found less immunogenic after vaccination as compared to other influenza subtypes^30,31^. For human evaluation, we will thus in line with the here presented ferret data use the tested high dose of 3.0 mg, but also a range of lower dose concentrations to get clinical information on the potential benefit of HLAII-specific targeting of HA. At least one of these should have a higher concentration than the 0.3 mg dose that here resulted in partial protection from disease. Our hypothesis is that the preclinically observed increased efficacy contributed by the HLAII-specific targeting unit used herein could be particularly important for obtaining rapid protection of the vaccinee against disease.

Targeting of antigen to different receptors on APC has consistently demonstrated improved immunogenicity upon vaccination^4–11,32–38^. Whereas targeting to chemokine receptors has demonstrated a particular benefit for induction of cellular immunity^6,9,10,35^, targeting the MHCII molecules is characterized by stronger antibody induction^5,6,11^. Importantly, MHCII-targeting of antigen also activates protective T-cell responses, providing an inherent breadth of protection against antigenically drifted variants of influenza. Importantly, we have demonstrated that the induced T-cell responses could prevent severe disease even before protective levels of antibodies were elicited^32^, and as such, rapidly confer protection against severe disease upon the new emergence of a pandemic threat.

Following DNA vaccination and translation to HLAII-targeted vaccine proteins, steering of HA to APC facilitates formation of an immunological synapse between the HLAII expressing APC and B cells. More specifically, the bivalent X-shaped vaccine proteins will bind HLAII molecules, likely making the remaining two HA arms easily accessible for B cell receptors (BCR). The bivalent display of HA promotes cross-linking of BCR, which is key for efficient activation^39,40^. The immunological synapse will, in turn efficiently promote formation of germinal centers to generate high affinity antibodies^40^. Here, we observed neutralizing antibodies already on day 12 after a single DNA vaccination in ferrets (Fig. 3D). These antibodies should, together with the induced T-cell responses, award some protection against disease early on, which could be key for protection in a scenario of mass spreading of a novel virus in the population.

Vaccine studies in ferrets are considered predictive of human responses to vaccines. Thus, the present results suggest that αHLAII-HS DNA vaccination against influenza H7N9 virus is safe and immunogenic in this pre-clinical animal model, and should be tested in humans. While ferrets are considered predictive of human responses for vaccines based on neutralizing antibodies as a correlate of protection, species differences nevertheless means that dose-response curves could be different in humans. As such, it will be important to also evaluate different doses of αHLAII-HS during the next stage of clinical evaluation (https://clinicaltrials.gov/study/NCT06046092).

## Methods

### Vaccine preparations

Codon optimized influenza HA from A/Shanghai/2/2013 (H7N9) was purchased from GenScript with flanking SfiI-sites and inserted into different plasmids encoding the previously described HLAII-specific vaccine format by subcloning^5,11^. For vaccination of mice, HA was inserted into pLNOH2^20^, pUMVC^23^, and NTC7482^24^. A clone of each of the resulting vaccine plasmids was inserted into competent *E. coli* by heat shock, and then expanded in animal-free growth medium. Vaccine plasmids were purified by Endofree Plasmid Mega Qiagen kit (Cat.no. 12381, Quiagen, the Netherlands), and dissolved in NaCl. The vaccine used for the ferret study was the technical batch from GMP production of a clinical grade DNA vaccine. In brief, pUMVC encoding the HLAII-targeted vaccine was grown in *E.coli* XL-1 Blue, and cells of the resulting bacterial pellet lysed by an RNase-free alkaline solution before two-step ultrafiltration. Purification was then performed in two chromatographic steps, ion exchange and hydrophobic interaction chromatography, followed by crossflow ultrafiltration/diafiltration and sterile filtration. The DNA vaccine was formulated in PBS pH 7.4. Acceptance criteria for the vaccine production included testing for appearance (e.g. visual check and weighing), pH, DNA concentration and purity (spectrophotometry), topology (>80% supercoiled plasmid DNA), endotoxin testing, and sterility testing.

### Sandwich ELISA

To assess in vitro protein expression of vaccine plasmids, ELISA plates (Costar 3590, Sigma-Aldrich, MO, US) were coated with 1 µg/ml of anti-human C_H_3 mAb (MCA878, AbD Serotec, CA, US), blocked, and incubated with supernatants collected 48 h after transient transfection of 293E cells (1 × 10^5^ cells/well) with the different vaccine plasmids in the presence of Lipofectamin2000 (11668-019, Invitrogen, Life Technologies, CA, US). Next, plates were incubated with biotinylated HP6017 (anti-human C_H_3) (409307, BioLegend, CA, US) (1 µg/ml) followed by Streptavidin alkaline phosphatase (1:5000) (7100-05, Southern Biotech, AL, US). Plates were developed using Phosphatase substrate (P4744-10G, Sigma Aldrich, MO, US) dissolved in substrate buffer, and read with a Tecan reader (Tecan, Switzerland) using the Magellan v5.03 program.

### Animals

#### Mice

The mice used in this study were BALB/c mice (Janvier Labs, France) and DQ2 transgenic mice^41^ on a BALB/c background, housed in a minimal disease unit at Oslo University Hospital. All mouse experiments were approved for ethics by the Norwegian Food Authority.

#### Ferrets

Outbreed (non-SPF and non-neutered) male ferrets (*Mustela putorius furo*), aged 6-7 months, were purchased from a commercial breeder (Highgate Farm, UK). Upon arrival they were confirmed to be negative for previous infections with circulating influenza viruses (in-house ELISA at WBVR, Lelystad, the Netherlands^42^) and Aleutian disease (counterimmunoelectrophoresis at CFE laboratory, Nederasselt, the Netherlands). Temperature loggers (DST micro T, Star-Oddi) recording the body temperature every 15 min were implanted intra-abdominally under general injection anesthesia (5 mg/kg ketamine and 0.1 mg/kg medetomidine hydrochloride) at 7 days prior to the start of the experiment (day 0). Meloxicam (0.2 mg/kg) was dosed as pre- and post-operative analgesic. Ferrets were identified by use of subcutaneous chips implanted by the breeder. All animals had ad libitum access to pelleted standard ferret food and tap water. Ferrets were group-housed per treatment group in pens (4.25 m^2^) on solid floors covered with wood shavings. Environmental enrichment of playing, climbing and hiding materials was provided.

The ferret trial was conducted in accordance with the Dutch Law on Animal Experiments (WoD) and the European legislations and guidelines (2010/63/EG and ETS 123). The current study was licensed by the Dutch Central Authority for Scientific Procedures on Animals (no. AVD401002015103) and approved by the Animal Welfare Body of Wageningen University and Research.

### Vaccination

#### Mice

Mice were anesthetized [0.1 mg/10 g body weight with cocktail of: Zoletil Forte (250 mg/ml) (Virbac France), Rompun (20 mg/ml), (Bayer Animal Health GmbH), and Fentanyl (50 µg/ml) (Actavis, Germany)] by intraperitoneal (i.p.) injection. Next, mice were shaved in the lower back region, and 12.5 µg plasmids in a 25 µl volume injected on each side (total DNA/mouse: 25 µg) immediately followed by skin electroporation (DermaVax, Cellectis, Paris, France).

#### Ferrets

Upon arrival, ferrets were allocated to either of three groups (*n* = 8) taking into account a similar median distribution of bodyweights. Two vaccinations of 3.0 mg DNA encoding αHLAII-HS (10 × 0.1 mL), 0.3 mg of DNA encoding αHLAII-HS (0.1 mL), or saline (0.1 mL) were administered intradermally after shaving of the upper thigh, and in the absence of anaesthesia, at day 0 and day 33 using the Tropis® intradermal needle-free injection system Jet Injector (NFIS) produced by PharmaJet (Golden, CO, USA)^35,37^. Following each vaccination, injection sites were observed for two days for swelling and redness.

### Serum ELISA

Blood from mice was harvested by puncture of the saphenous vein. For ferrets, blood samples were collected from the *Vena cava cranialis* under general injection anaesthesia, as described above. Sera were isolated by centrifugation and responses of individual animals assayed by ELISA. In brief, ELISA plates (Costar 3590) were coated with 0.5 µg/ml rec. HA [A/Shanghai/1/2013 (H7N9): 40104-V08B; A/Shanghai/2/2013 (H7N9): 40239-V08B, both from Sino Biological Inc.], blocked with 1% BSA, and incubated with serially diluted serum samples. HA-specific antibodies in mice were detected with alkaline phosphatase conjugated goat anti-mouse IgG (A1418, Sigma-Aldrich), and with alkaline phosphatase conjugated anti-ferret IgG (LS-C61240, LSBio, WA, US) in ferrets. Plates were then developed with phosphatase substrate (P4744, Sigma Aldrich), and analysed as described above. For all serum ELISAs, endpoint titers were determined as the last serum dilution with OD_405_ above background (mean absorbance from NaCl vaccinated mice added 5 times the standard error of the mean for the group).

### Haemagglutination inhibition (HI) assay

Sera collected as above were analyzed in an HI assay against 3–6 Haemagglutination Units (HAU) of BPL-inactivated whole-virus A/Anhui/1/2013 (H7N9) (ref. NIBSC 16/238), using 1% chicken erythrocytes. Prior to testing, serum samples had been heat-inactivated (56 °C), incubated overnight (ON) with Receptor Destroying Enzyme (37 °C), and pre-treated with chicken erythrocytes for 1 hr (4 °C). Starting with a 1:10 dilution, sera were 2-fold serially diluted and tested in duplicate. The HI titer was defined as the reciprocal value of the serum dilution that resulted in inhibition of agglutination in 50% of the duplicate wells. Negative samples were assigned a value of 5.

### Pseudotyped virus neutralization assay

Pseudotyped virus neutralization assay was performed as previously described^43^. In brief, 3 × 10^3^ MDCK cells were seeded in each well of a 96-well culture plate (Corning, NY, USA) and incubated ON at 37°C in 5% CO_2_. Serially diluted serum samples, that had been pre-incubated with HA pseudotypes from A/Shanghai/1/2013 (H7N9), A/Shanghai/2/2013 (H7N9), A/chicken/Italy/13474/1999 (H7N1), A/chicken/Chile/4322/2002 (H7N3), A/Guandong/17SF003/2016 (H7N9), and A/Vietnam/1203/2004 (H5N1) (negative control) [2000–200,000 relative luciferase activity (RLA)] at 37 °C in 5% CO_2_ for 1 h, were added to the cells for 72 h incubation at 37°C in 5% CO_2_. RLA was measured by a BrightGlo Luciferase assay according to the manufacturer’s instructions (Promega, Madison, WI, USA). The percentage of inhibition was calculated by: (RLA in pseudotypes and medium control—RLA in pseudotypes and immune serum at a given dilution)/(RLA in pseudotypes and medium control). The data were fitted to a sigmoidal dose response curve using GraphPad Prism 6 software, and IC80 were determined from those data sets.

### Interferon gamma ELISPOT

Peripheral blood mononuclear cells (PBMC) from ferrets were isolated from 1:1 PBS-diluted heparinized blood by density gradient centrifugation (1:1 LymphoPrep 1114547 and Lympholyte-M CL5035, Sanbio) at 800 x g. After washing in PBS and incubation with ACK buffer to lyse residual erythrocytes, the final cell pellet was resuspended in PBS and kept ON at 4 °C. On the next day, 5 × 10^5^ cells per well on U-shaped cell culture plates were incubated with 5 µg/ml of BPL inactivated A/Anhui/1/2013 (H7N9) (NIBSC 16/238), or 5 µg/ml of HA protein from A/Shanghai/2/2013 (H7N9) (Sino Biological, 40239-V08H) for 20-24 hours at 37 °C in a 5% CO_2_ incubator. Cells incubated with either Concanavalin A (20 µg/ml) or medium were taken along as positive and negative control for T cell stimulation, respectively. On the next day, cells were transferred to V-shaped plates, re-incubated with fresh stimuli on pre-coated Ferret IFNγ-ELISpot plates (3112-4APW-2, Mabtech), and incubated for 20–24 h at 37 °C in a 5% CO_2_ incubator. The day after, plates were stained according to the ELISpot kit protocol, air-dried and read on the Immunospot^®^ Analyzer (CTL). Background was corrected by subtraction of medium stimulated spot counts from antigen specific stimulated spot counts.

### Virus and viral challenges

#### Mice

Mice were anesthetized as previously described and inoculated with 5xLD_50_ mouse adapted A/turkey/Italy/3889/1999 (H7N1) in 10 µl/nostril. Mice were monitored for weight loss, and mice that reached the humane endpoint of >20% weight loss were euthanized by cervical dislocation.

#### Ferrets

Isolate A/Anhui/1/2013 (H7N9) was obtained from the National Institute for Biological Standards and Control (NIBSC, ref. 13/104) under the conditions of the WHO Pandemic Influenza Preparedness (PIP) Framework. The virus batch used in the ferret study was passaged once on Madin-Darby Canine Kidney (MDCK) cells at Wageningen Bioveterinary Research.

Ferrets were challenged with 10^5^ TCID_50_ of Anhui/1/2013 (H7N9) by intra-tracheal administration in a volume of 3 ml. The inoculation of challenge virus was performed under general injection anaesthesia as described above. Four days after viral challenge, the experiment was ended by subjecting the animals to necropsy. From the day of challenge until necropsy, body temperatures were recorded by the intraabdominal temperature loggers and ferrets were clinically observed twice daily to assess the severity of depression, nasal discharge and respiratory distress, with a score of 0, 1, 2 or 3 (corresponding to absent, mild, moderate or severe) according to scoring table provided as Supplementary Fig. 8. Ferrets were also scored for sneezing/coughing, skin changes, edema, neurological symptoms, and diarrhea, but no clinical signs were observed for either of these. Throat swabs and body weights were collected just prior to challenge and on days 2, 3, and 4 under general injection anaesthesia (as described above). After collection, swabs were placed in 2 mL of 3% tryptase phosphate buffer supplemented with antibiotics, vortexed, and stored at <−70 °C to assess viral loads.

### Gross- and histopathology of ferret lungs

At necropsy, ferrets were sacrificed by cardiac bleeding under general injection anaesthesia. Upon removal, lungs were weighed, and areas of consolidated lung tissue were captured on a sketch to calculate the total affected area. Samples of the right lung, the trachea middle section and the right half of nasal turbinates were stored at −70 °C to assess tissue viral loads. Samples of the left lung (inflated by 10% buffered formalin) and left nasal turbinates were fixed in 10% buffered formalin for histopathological analysis.

Formalin-fixed tissues were embedded in paraffin, and tissue sections stained with hematoxylin and eosin (HE) stain. For immunohistochemical staining (IHC), tissue sections were dewaxed, endogenous peroxidase was inhibited by methanol/H_2_O_2_ treatment followed by treatment with protease 24 (0.1 mg/ml, Sigma), and blocking using 10% normal goat serum. Thereafter, slides were incubated with a mAb against influenza virus A nucleoprotein (HB 65, H16-L10-4R5, ATCC), with a dilution of 1:100 for 45 min at room temperature (RT), followed by incubation with a secondary antibody (mouse Envision peroxidase polymer Dako K4001). Binding was visualized by 3,3’-diaminobenzidine (DAB) (Dako K3468) as substrate. Slides were counterstained with haematoxylin. As staining controls, infected and non-infected lung tissue of ferrets were used, and the primary antibody was replaced with an isotype control. Histopathological analysis of HE stained slides and IHC-stained slides was performed in a blinded manner. The severity of histopathologic changes was semi quantitatively graded from score 0 (no changes), score 1 (focal or multifocal mild changes), score 2 (multifocal moderate changes), score 3 (multifocal severe changes), and score 4 (coalescing, very severe changes); the findings when examining both lung specimens were combined into one score. The IHC stained slides were examined for the presence of brown stained, virus-antigen positive cells and the extension of viral expression was semi-quantitatively graded: score 0 (negative), score 1 (focal or multifocal, <3 foci, sparse staining), score 2 (moderate staining, multifocal, >3 foci), score 3 (abundant staining, multifocal to coalescing) or score 4 (excessive staining, diffuse staining).

### Virus quantification in ferret throat swabs and tissues

Upon freezing, lung, trachea, and nasal turbinate tissue samples were weighed and homogenized in at least 5 mL of cell culture medium in an Ultra-Turrax^®^ homogenizer. Total nucleic acids were extracted from 200 μL sample of the tissue homogenates and throat swab samples using the MagNA Pure 96 DNA and Viral NA Small Volume Kit® and MagNA Pure LC® 96 isolation robot workstation (Roche). The influenza M-gene was detected by real-time RT-PCR with a primer/taqman probe mix (forward primer CTTCTAACCGAGGTCGAAACGTA, reverse primer CACTGGGCACGGTGAGC, and Taqman probe 6FAM– TCAGGCCCCCTCAAAGCCGA) using the QuantiFast Multiplex Kit (Qiagen) according to the manufacturer’s instructions. All assays included reverse transcription of RNA into cDNA and were run on the Mx3005P® Strategene qPCR system (Qiagen) under optimized cycling conditions (45 cycles). A calibration curve consisting of a serial dilution of H7N9 with known amounts of infectious virus (TCID_50_) was included in each PCR analysis. PCR threshold cycle (Ct) values were converted to PCR equivalent titers expressed as log_10_ TCID_50_ per mL (for swab samples) or per gram (for tissue samples). Negative samples were assigned a value of 0 log_10_.

Lung homogenates (10% w/v suspensions) were analysed by end-point titration on MDCK cells. Briefly, 10-fold serial dilutions of the samples (starting with 1:10) were added to MDCK cell cultures and tested in duplicate. After two days of incubation at 37 °C, the cells were permeabilized, fixed in 4% paraformaldehyde and subjected to immunoperoxidase staining using NP-specific mAb HB65 (WBVR, Lelystad). Virus titers were calculated according to Reed and Muench, and expressed as 50% tissue culture infectious dose (TCID_50_/mL). Based on tissue weights, viral titers were converted to TCID_50_/gram. Negative samples were assigned a value of 1.8 log_10_ TCID_50_/gram which was 1 log_10_ beyond the detection limit of the assay.

### Statistical analysis

Multiple comparisons were analysed with two-way ANOVA and Tukey’s multiple comparison test, to test for statistical significance of differences. Comparisons of two groups were analysed with the nonparametric Kruskal–Wallis test (or one-way ANOVA on ranks) followed by a Wilcoxon signed-rank test to test for statistical significance of differences between groups on a specific time-point. Survival was assessed by the Mantel-Cox test of Kaplan Meyer curves. In all tests, *p* < 0.05 was considered statistically significant. All statistics for mouse data were done using GraphPad Prism software, version 9 (GraphPad Prism, San Diego, CA), whereas statistics for ferret data were performed using R (version 4.3.2).

## Supplementary information

Supplementary Information

## Data Availability

Raw data from all experiments will be made available upon request to the corresponding author.
