# Supplementary material for: An HLAII-targeted DNA vaccine against influenza H7N9 protected mice and ferrets from a virus challenge
Source: NPJ Vaccines. 2025 Dec 24;11:20. doi: 10.1038/s41541-025-01341-4 (PMC12824389; doi:10.1038/s41541-025-01341-4)
Supplement: Supplementary file 1 — Supplementary Information [file 41541_2025_1341_MOESM1_ESM.pdf]

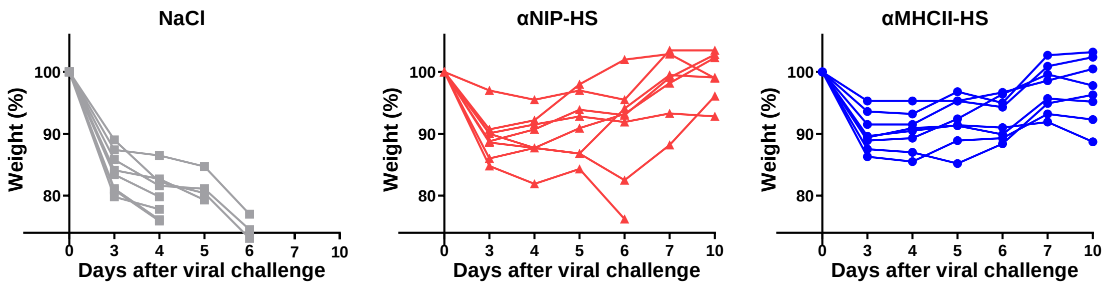

Supplemental Figure 1. Weight following viral challenge with influenza H7N1 in mice. BALB/c mice (n=8/group) were vaccinated once i.d. with 25µg DNA encoding the indicated vaccines. At day 42 post vaccination, mice were challenged with a 5xLD50 dose of influenza A/turkey/Italy/3889/1999 (H7N1) virus and monitored for weight. (See also Figure 1, C-E.)

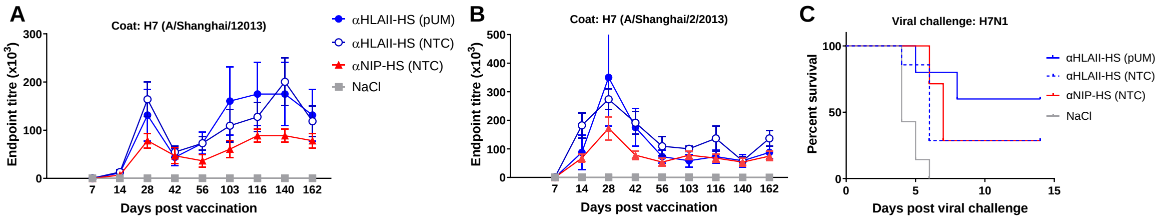

Supplemental Figure 2. Vector comparison in mice II. DQ2 transgenic mice [n=7/group, except for αHLAII-HS (pUM) where there were 5/group] were vaccinated once i.d. with 25µg DNA encoding the indicated vaccines. Sera from individual vaccinated mice were evaluated for IgG at different timepoints in ELISA against A/Shanghai/1/2013 (H7N9) (A) and A/Shanghai/2/2013 (H7N9) (B). (C) At week 25 post vaccination, mice were challenged with a 10xLD50 dose of influenza A/turkey/Italy/3889/1999 (H7N1) virus. Depicted is survival as defined by the humane endpoint (20% weight loss).

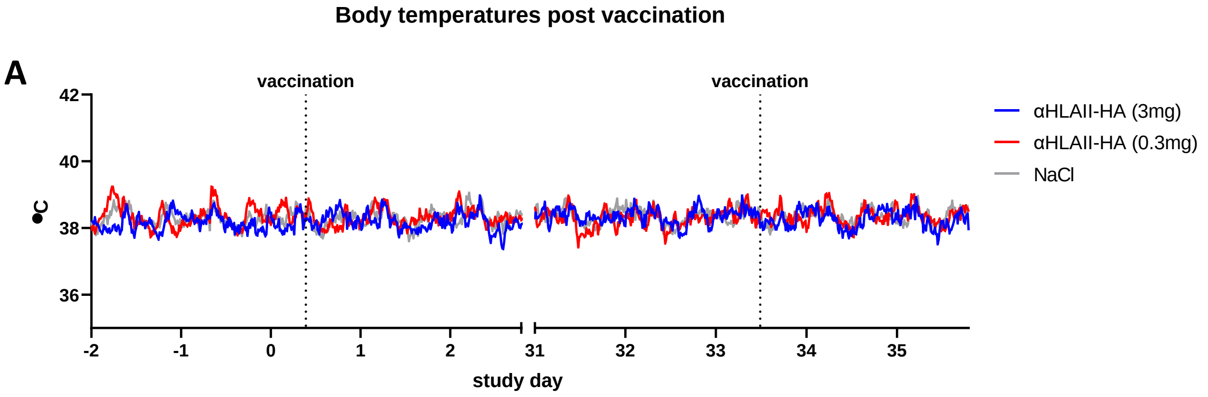

B

Group	Ferret / study day	D0	D1	D2	D33	D34	D35
		post 1st vaccination			post 2nd vaccination		
T01 HLAII-HS 1mL (3.0mg)	491	0	1 (1 spot: 2)	1 spot: 2	0	1 spot: 2	1 spot: 1 & 1 spot: 2
	044	0	1	0	0	1 (3 spots: 2)	2 spots: 1
	356	0	1	1 (brown colour)	0	1 (2 spots: 2	0
	014	0	1	0	0	1	0
	422	0	1	0	0	1	0
	765	0	1	0	0	1 (2 spots: 2)	1 spot: 1
	802	0	1 (1 spot: 2)	1 spot: 1	0	1 spot: 2 & 1 spot: 3	2 spots: 2
	395	0	1 (2 spots: 2)	1 spot: 1 & 1 spot: 2	0	1 (1 spot: 2)	1 spot: 1
T02 HLAII-HS 0.1mL (0.3mg)	167	0	0	0	0	0	0
	298	0	1	0	0	0	0
	495	0	0	0	0	0	0
	314	0	1	0	0	0	0
	481	0	1	0	0	0	0
	567	0	0	0	0	0	0
	708	0	0	0	0	0	0
	898	0	1	0	0	1	0
T03 PBS	264	0	0	0	0	0	0
	557	0	0	0	0	0	0
	730	0	0	0	0	0	0
	309	0	1	0	0	1	0
	534	0	0	0	0	0	0
	630	0	1	0	0	0	0
	565	0	0	0	0	0	0
	318	0	0	0	0	0	0

Supplemental Figure 3. Temperature and injection site reactions post vaccination. Ferrets were vaccinated as indicated on days 0 and 33. (A) Temperatures were continuously monitored by inserted transponders. (B) At day 1 and 2 post vaccination, the site of vaccination was observed for swelling and redness (score 0 absent; score 1 mild, score 2 moderate).

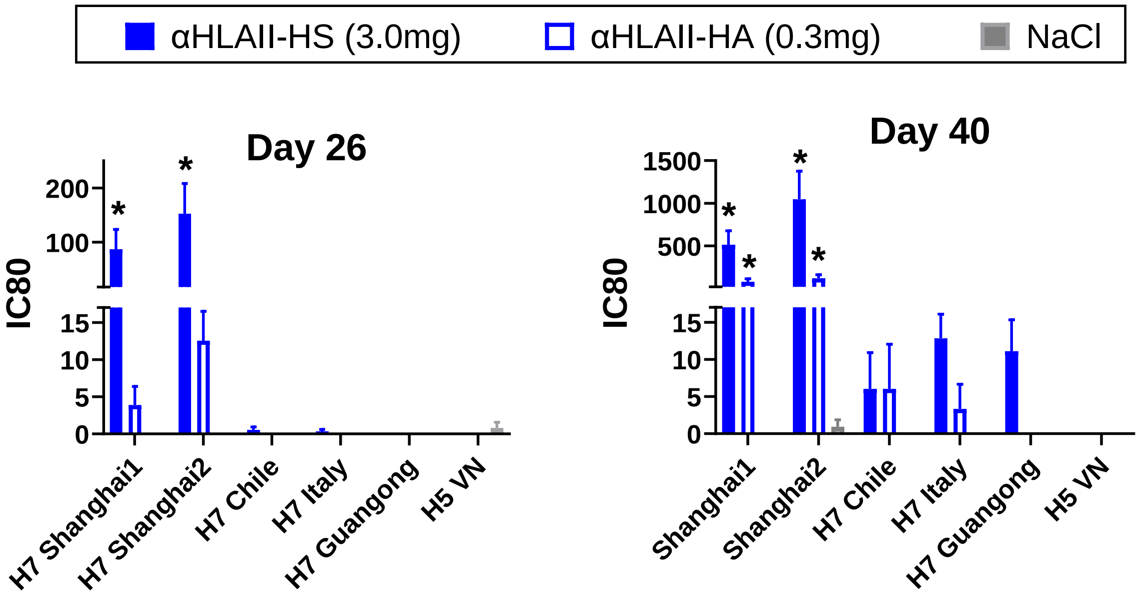

Supplemental Figure 4. Pseudotype neutralization assay – cross reactive responses. Sera after one (day 26) and two DNA vaccinations (day 40) were evaluated in a pseudotype neutralization assays against A/Shanghai/1/2013 (H7N9), A/Shanghai/2/2013 (H7N9), A/chicken/Chile/4322/2002 (H7N3), A/chicken/Italy/13474/1999 (H7N1), A/Guandong/17SF003/2016 (H7N9), and A/Vietnam/1203/2004 (H5N1) (negative control). *p<0.02 as compared to NaCl, One-Way ANOVA and Tukey's multiple comparison test.

```
A/turkey/Italy/3889/1999      DKICLGHHAVSNGTKVNTLTERGVEVVNATETVERTNVPRICSKGKRTVDLGQCGLLGTI    60
A/chicken/Italy/13474/99      -KICLGHHAVSNGTKVNTLTERGVEVVNATETVERTNVPRICSKGKRTVDLGQCGLLGTI    59
A/Guandong/17SF003/2016       DKICLGHHAVSNGTKVNTLTERGVEVVNATETVERTNTPRICSKGKRTVDLGQCGLLGTI    60
A/Shanghai/2/2013             DKICLGHHAVSNGTKVNTLTERGVEVVNATETVERTNIPRICSKGKRTVDLGQCGLLGTI    60
A/Shanghai/1/2013             DKICLGHHAVSNGTKVNTLTERGVEVVNATETVERTNIPRICSKGKRTVDLGQCGLLGTI    60
                               ************************************ **********************

A/turkey/Italy/3889/1999      TGPPQCDQFLEFSADLIIERREGSGVCYPGKFVNEEALRQILRESGGIDKETMGFTYSGI    120
A/chicken/Italy/13474/99      TGPPQCDQFLEFSADLIIERREGSDVCYPGKFVNEEALRQILRESGGIDKE-AGFTYSGI    118
A/Guandong/17SF003/2016       TGPPQCDQFLEFSADLIIERREGSDVCYPGKFVNEEALRQILRESGGIDKEPMGFTYNGI    120
A/Shanghai/2/2013             TGPPQCDQFLEFSADLIIERREGSDVCYPGKFVNEEALRQILRESGGIDKEAMGFTYSGI    120
A/Shanghai/1/2013             TGPPQCDQFLEFSADLIIERREGSDVCYPGKFVNEEALRQILRESGGIDKEAMGFTYSGI    120
                              ************************.**************************  ****.**

A/turkey/Italy/3889/1999      RTNGTTSACRRLGSSFYAEMKWLLSNTDNAAFPQMTKSYKNTRKDPALIIWGIHHSGSTT    180
A/chicken/Italy/13474/99      RTNGTTSTCRRSGSSFYAEMKWLLSNTDNAAFPQMTKSYKNTRKDPALIIWGIHHSGSTT    178
A/Guandong/17SF003/2016       RTNGVTSACRRSGSSFYAEMKWLLSNTDNAAFPQMTKSYKNTKESPAIIVWGIHHSVSTA    180
A/Shanghai/2/2013             RTNGATSACRRSGSSFYAEMKWLLSNTDNAAFPQMTKSYKNTRKSPALIVWGIHHSVSTA    180
A/Shanghai/1/2013             RTNGATSSCRRSGSSFYAEMKWLLSNTDNAAFPQMTKSYKNTRKNPALIVWGIHHSGSTA    180
                              ****.**:*** ******************************::.**:*:****** **:

A/turkey/Italy/3889/1999      EQTKLYGSGNKLITVGSSNYQQSFVPSPGARPQVNGQSGRIDFHWLMLNPNDTVTFSFNG    240
A/chicken/Italy/13474/99      EQTKLYGSGNKLITVGSSNYQQSFVPSPGERPQVNGQSGRIDFHWLMLNPNDTVTFSFNG    238
A/Guandong/17SF003/2016       EQTKLYGSGNKLVTVGSSNYQQSFVPSPGARPQVNGQSGRIDFHWLILNPNDTVTFSFNG    240
A/Shanghai/2/2013             EQTKLYGSGNKLVTVGSSNYQQSFVPSPGARPQVNGLSGRIDFHWLMLNPNDTVTFSFNG    240
A/Shanghai/1/2013             EQTKLYGSGNKLVTVGSSNYQQSFVPSPGARTQVNGQSGRIDFHWLMLNPNDTVTFSFNG    240
                              ************:**************** * **** *********:*************

A/turkey/Italy/3889/1999      AFIAPDRASFLRGKSMGIQSGVQVDANCEGDCYHSGGTIISNLPFQNINSRAVGKCPRYV    300
A/chicken/Italy/13474/99      AFIAPDRASFLRGKSMGIQSGVQVDANCEGDCYHSGGTIISNLPFQNINSRAVGKCPRYV    298
A/Guandong/17SF003/2016       AFIAPDRASFLRGKSMGIQSRVQVDANCEGDCYHSGGTIISNLPFQNIDSRAVGKCPRYV    300
A/Shanghai/2/2013             AFIAPDRASFLRGKSMGIQSGVQVDANCEGDCYHSGGTIISNLPFQNIDSRAVGKCPRYV    300
A/Shanghai/1/2013             AFIAPDRASFLRGKSMGIQSGVQVDADCEGDCYYSGGTIISNLPFQNIDSRAVGKCPRYV    300
                              ******************** *****:******:**************:***********
```

```
A/turkey/Italy/3889/1999      KQESLLLATGMKNVPEVPKG----RGLFGAIAGFIENGWEGLIDGWYGFRHQNAQGEGTA    356
A/chicken/Italy/13474/99      KQESLLLATGMKNVPEIPKGSRVRRGLFGAIAGFIENGWEGLIDGWYGFRHQNAQGEGTA    358
A/Guandong/17SF003/2016       KQRSLLLATGMKNVPEVPKRKRTARGLFGAIAGFIENGWEGLIDGWYGFRHQNAQGEGTA    360
A/Shanghai/2/2013             KQRSLLLATGMKNVPEIPK----GRGLFGAIAGFIENGWEGLIDGWYGFRHQNAQGEGTA    356
A/Shanghai/1/2013             KQRSLLLATGMKNVPEIPK----GRGLFGAIAGFIENGWEGLIDGWYGFRHQNAQGEGTA    356
                              **.*************:**     ************************************

A/turkey/Italy/3889/1999      ADYKSTQSAIDQVTGKLNRLIEKTNQQFELIDNEFTEVEKQIGNVINWTRDSMTEVWSYN    416
A/chicken/Italy/13474/99      ADYKSTQSAIDQVTGKLNRLIEKTNQQFELIDNEFTEVEKQIGNVINWTRDSMTEVWSYN    418
A/Guandong/17SF003/2016       ADYKSTQSAIDQITGKLNRLIAKTNQQFKLIDNEFNEVEKQIGNVINWTRDSITEVWSYN    420
A/Shanghai/2/2013             ADYKSTQSAIDQITGKLNRLIEKTNQQFELIDNEFNEVEKQIGNVINWTRDSITEVWSYN    416
A/Shanghai/1/2013             ADYKSTQSAIDQITGKLNRLIEKTNQQFELIDNEFTEVEKQIGNVINWTRDSITEVWSYN    416
                              ************:******** ******:******.****************:*******

A/turkey/Italy/3889/1999      AELLVAMENQHTIDLADSEMNKLYERVKRQLRENAEEDGTGCFEIFHKCDDDCMASIRNN    476
A/chicken/Italy/13474/99      AELLVAMENQHTIDLTDSEMNKLYERVKRLLRENAEEDGTGCFEIFHKCDDDCMASIRNN    478
A/Guandong/17SF003/2016       AELLVAMENQHTIDLADSEMDKLYERVKRQLRENAEEDGTGCFEIFHKCDDDCMASIRNN    480
A/Shanghai/2/2013             AELLVAMENQHTIDLADSEMDKLYERVKRQLRENAEEDGTGCFEIFHKCDDDCMASIRNN    476
A/Shanghai/1/2013             AELLVAMENQHTIDLADSEMDKLYERVKRQLRENAEEDGTGCFEIFHKCDDDCMASIRNN    476
                              ***************:****:******** ******************************

A/turkey/Italy/3889/1999      TYDHSKYREEAMQNRIQIDPVKLSSGYKDVILWFSFGA    514
A/chicken/Italy/13474/99      TYDHSKYREEAMQNRIQIDPVKLSSGYKDVILWFSFGA    516
A/Guandong/17SF003/2016       TYDHRKYREEAMQNRIQIDPVKLSSGYKDVILWFSFGA    518
A/Shanghai/2/2013             TYDHSKYREEAMQNRIQIDPVKLSSGYKDVILWFSFGA    514
A/Shanghai/1/2013             TYDHSKYREEAMQNRIQIDPVKLSSGYKDVILWFSFGA    514
                              **** *********************************
```

Supplemental Figure 5. Sequence alignment: HA from relevant H7N9 influenza viruses. Sequences of vaccine inserted HA from relevant influenza H7N9 viruses were evaluated using a CLUSTAL O (1.2.4) multiple sequence alignment.

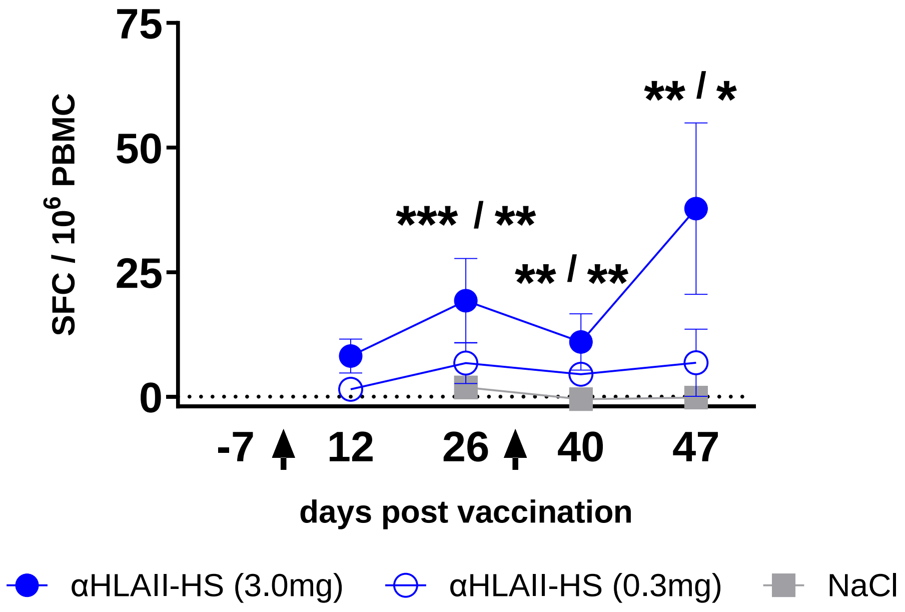

Supplemental Figure 6. IFNγ secreting cells following vaccination. Ferrets were vaccinated twice as indicated, and blood collected longitudinally for IFNγ ELISpot. PBMC were stimulated *in vitro* with inactivated A/Anhui/1/2013 (H7N9) influenza virus. Shown are counts (mean +/- 95% CI) of IFNγ secreting cells. On day 12, n=5 of 3 mg αHLAII-HS vaccinated ferrets and n=2 of 0.3 mg αHLAII-HS vaccinated ferrets. On day 40, n=6 of 0.3 mg αHLAII-HS vaccinated ferrets and n=7 of NaCl vaccinated ferrets. For days 26 and 47: n=8/group. Kruskal Wallis per timepoint followed by Wilcoxon test for pair-wise comparison: ns: non-significant, **p< 0.01 as compared to NaCl. Left of "/ " = results of αHLAII-HS (3mg) , right results of αHLAII-HS (0.3mg) .

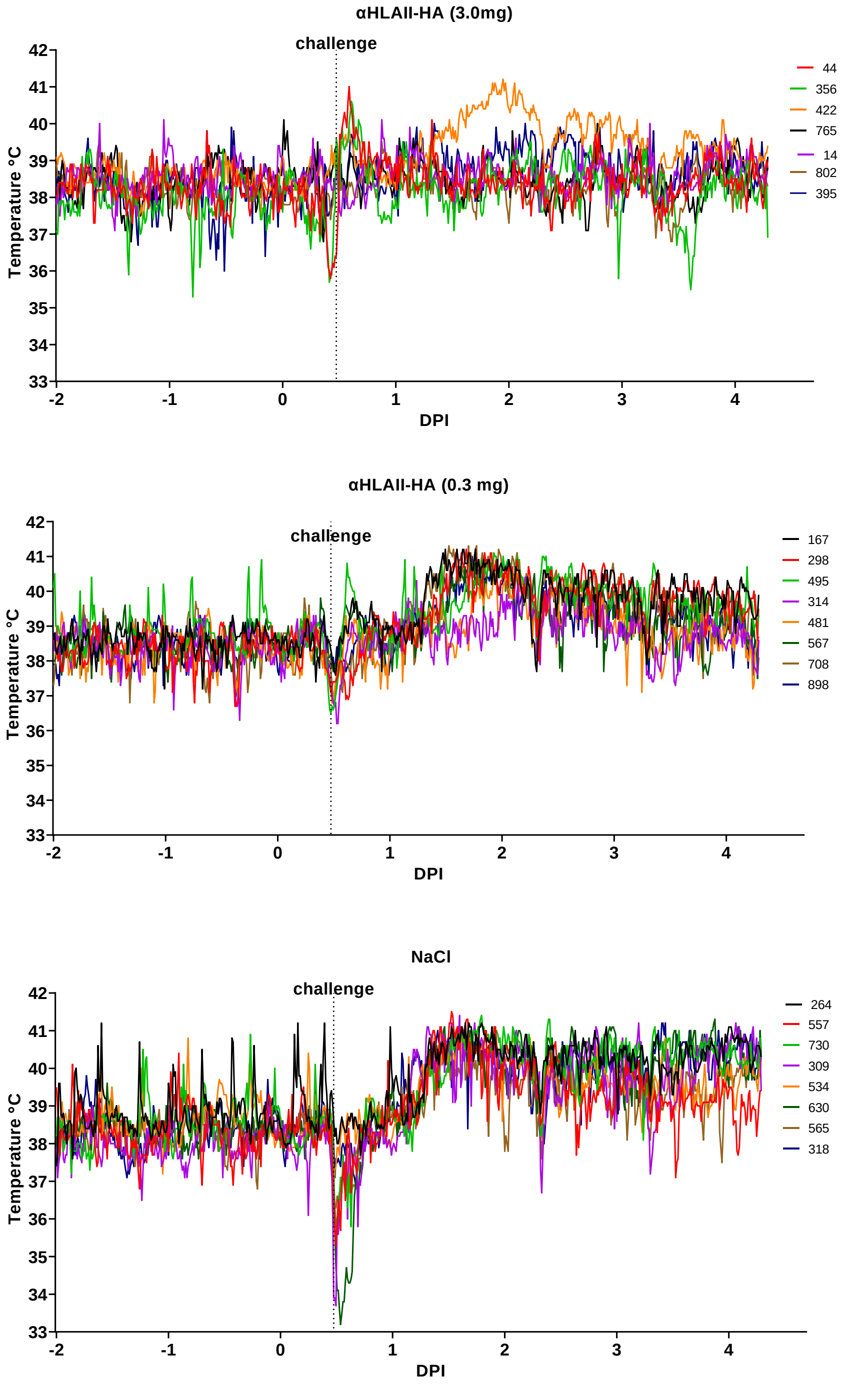

Supplemental Figure 7. Individual mean body temperatures in ferrets after viral influenza challenge. Temperature loggers were surgically transplanted intra-abdominally for continuous measurement of individual mean body temperatures (n=8 ferrets/group). Due to the malfunctioning of one transponder, n=7 for the 3.0 mg αHLAII-HS group**.**

Supplemental Figure 8. Scoring table for clinical evaluations of ferrets after viral influenza H7N9 challenge (severity score 1 = mild, score 2 = moderate, score 3 = severe).

	Symptom	Score
Respiratory and stomach/ intestine symptoms	Sneezing	1
	Nasal discharge	1
	Diarrhea	1
Breathing	Normal breathing	0
	Impaired breathing	1
	Pumping and heavy breathing	2
Activity	Alert	0
	Alert when stimulated	1
	Inactive	2
	Lethargic	3
Neurological symptoms	Non	0
	Mild ataxia – uncoordinated movements	1
	Ataxia – stiff or bend neck	2
	Severe ataxia – complete paralysis of hind limb	3
